
JOURNAL INFORMATION
==============================
Journal ID: NuR

Natur und Recht

ISSN: 0172-1631
EISSN: 1439-0515

ARTICLE INFORMATION
==============================
PMCID: PMC8054688
DOI: 10.1007/s10357-021-3828-0
Article ID: 3828
Article version: 1
Subjects: Berichte

Tagungsbericht zur 3. Promovierendenkonferenz Umwelt und Recht, 24. und 25.9.2020

Iwanczik Fabian
grid.10388.32 0000 0001 2240 3300 Rheinische Friedrich-Wilhelms-Universität Bonn, Bonn, Deutschland
Electronic publication date: 2021 Apr 19
Print publication date: 2021
Volume: 43
Issue: 4
First page: 256
Last page: 258
Copyright: © Der/die Autor(en) 2021
License: Open Access Dieser Artikel wird unter der Creative Commons Namensnennung 4.0 International Lizenz veröffentlicht, welche die Nutzung, Vervielfältigung, Bearbeitung, Verbreitung und Wiedergabe in jeglichem Medium und Format erlaubt, sofern Sie den/die ursprünglichen Autor(en) und die Quelle ordnungsgemäß nennen, einen Link zur Creative Commons Lizenz beifügen und angeben, ob Änderungen vorgenommen wurden.
Die in diesem Artikel enthaltenen Bilder und sonstiges Drittmaterial unterliegen ebenfalls der genannten Creative Commons Lizenz, sofern sich aus der Abbildungslegende nichts anderes ergibt. Sofern das betreffende Material nicht unter der genannten Creative Commons Lizenz steht und die betreffende Handlung nicht nach gesetzlichen Vorschriften erlaubt ist, ist für die oben aufgeführten Weiterverwendungen des Materials die Einwilligung des jeweiligen Rechteinhabers einzuholen.
Weitere Details zur Lizenz entnehmen Sie bitte der Lizenzinformation auf http://creativecommons.org/licenses/by/4.0/deed.de.
License URL: https://creativecommons.org/licenses/by/4.0/

issue-copyright-statement: © Springer-Verlag GmbH Deutschland, ein Teil von Springer Nature 2021

==============================

**Fabian Iwanczik**

Die „Promovierendenkonferenz Umwelt und Recht“ (ProKUR) ist eine zweitägige Veranstaltung, auf welcher sich Promovierende, deren Themen umweltrechtliche Bezüge aufweisen, gegenseitig kennenlernen, vernetzen und diskutieren können. Das Stadium des Promotionsvorhabens spielt für die Teilnahme keine Rolle: Von einem bloßen Promotionsinteresse bis hin zu einem fast einreichungsreifen Manuskript sind Personen aus allen Stadien willkommen und vertreten. Niedrigschwellig können (Promotions-)Themen vorgestellt sowie methodische und organisatorische Fragen diskutiert werden. Die Teilnehmer geben gegenseitig Anmerkungen und Hilfestellungen, beispielsweise über Gliederungsentwürfe und Exposés, die verschiedenen Finanzierungsmöglichkeiten oder den Umgang mit Literaturverwaltungsprogrammen. Aber auch für aktuelle Fragen des Umweltrechts besteht ein Raum zur Diskussion. Ein solcher Austausch zwischen den Doktoranden erfolgt mitunter schon innerhalb des Arbeitsalltags oder im Rahmen spezieller Veranstaltungen an einzelnen Instituten oder Universitäten. Die ProKUR bietet darüber hinaus einen Rahmen für einen ortsübergreifenden Austausch mit vielfältigen Gesprächspartnern von anderen Universitäten. Auch externe Doktoranden erhalten so eine einfache Möglichkeit mit anderen Doktoranden in Kontakt zu kommen.

Nach Leipzig (2018) und Bremen (2019) richtete dieses Jahr ein Organisationsteam aus Münster die nunmehr dritte Auflage der ProKUR aus. Wegen der Covid-19-Pandemie fand die ProKUR erstmalig als Online-Veranstaltung statt, gewissermaßen als „eProKUR“. 51 Teilnehmer, vor allem aus Deutschland, aber auch aus Österreich, den Niederlanden und Brasilien, schalteten sich aus ihren Büros, Küchen, Arbeits- und Wohnzimmern zu.

Nach einer Begrüßung durch das Organisationsteam richtete Prof. Dr. *Sabine Schlacke* (Universität Münster) ein Grußwort an die Teilnehmer. Sie lobte das „vielfältige Programm“, welches „neben den medienwirksamen Themen wie Klimaschutz“ auch weitere Herausforderungen des Umweltrechts behandeln würde. Ihr Wunsch, dass die Teilnehmer eine „konstruktive Atmosphäre“ und „ein miteinander, nicht gegeneinander“ haben würden, sollte sich während der zwei Tage bewahrheiten.

Fabian Iwanczik, Wissenschaftlicher Mitarbeiter und Doktorand im Rahmen des Kompetenznetzwerks „Zukunftsherausforderungen des Umweltrechts“ (KomUR) von Prof. Dr. Dr. Durner LL.M., Rheinische Friedrich-Wilhelms-Universität Bonn, Bonn, Deutschland

**1. Vorträge**

Den ersten Vortrag hielt *Lorenz Strauch* (FU Berlin) über „Territorialität als Herausforderung für ein internationales Bodenschutzregime". In einer interdisziplinären Einleitung warf er einen Blick auf andere bodenbezogene Disziplinen wie die Geologie, bevor er auf die Bedeutung des Bodens in der Rechtswissenschaft einging, etwa im Rahmen der Jellinek'schen Drei-Elemente-Lehre. Viele Bodenfunktionen hätten eine grenzüberschreitende Wirkung, sodass ein überstaatliches Regelungsregime eigentlich angezeigt sei. Insbesondere auf völkerrechtlicher Ebene sei der Normbestand jedoch fragmentarisch und bestehe häufig nur aus soft law, beispielsweise SDG 15.3 der UN. Sodann widmete sich *Julian Senders* (Universität Jena) mit „Zusammenwirken quellen- und gebietsorientierter Regelungsansätze im verkehrsbezogenen Luftqualitätsrecht" einem aktuellen und medienwirksamen Thema. Zunächst wurden die Auswirkungen von Luftschadstoffen auf die Umwelt und die menschliche Gesundheit anschaulich erläutert. Anschließend stellte er dar, auf welche Weise in seiner Dissertation zum Beispiel das Fahrzeuggenehmigungsrecht mit dem gebietsbezogenen Luftqualitätsrecht verknüpft werden soll. Dabei wurden Parallelen zu anderen Rechtsgebieten wie dem gebietsbezogenen Wasserrecht aufgezeigt.

Die beiden nachfolgenden Vorträge widmeten sich Themen mit Bezug zum Bereich Energie. Der Vortrag von *Ann-Katrin Schneider* (Universität Würzburg) zum ebenfalls aktuellen Thema „Der ‚$CO_2$-Preis' in den Sektoren Wärme und Verkehr als Antwort auf die Klimakrise?" „ließ sogar das Finanzverfassungsrecht anschaulich werden", so ein Teilnehmer. Im Zentrum stand die Einordnung des Brennstoffemissionshandelsgesetzes in das Spektrum möglicher öffentlicher Abgaben sowie die sachliche Rechtfertigung dafür. *Roman Weidinger* (Universität Greifswald) präsentierte sein Promotionsvorhaben „Erneuerbare-Energien-Erzeugungsplanung – Ausgestaltung eines Fachplanungsregimes für Erneuerbare-Energien-Anlagen" (EE-Anlagen). Angesichts der Ausbaukrise von EE-Anlagen wurden grundlegende Fragen aufgeworfen, etwa die Wahl zwischen staatlicher Planung und Marktmechanismen. Auch generelle Planungshindernisse wurden angesprochen. Anknüpfend an spezielle Planungsregime einzelner Infrastrukturbereiche wie dem Netzausbau mündeten die präsentierten Überlegungen in dem Design eines neuen Fachplanungsregimes für EE-Anlagen, welches einerseits Allokationsfragen und Flächennutzung miteinander verknüpfen soll und andererseits zwischen Bedarfs- und Verwirklichungsplanung trennt.

*Jula Zenetti* (UFZ Leipzig) hielt einen Vortrag über „Eigenrechte der Natur". Zu Beginn stellte sie die Entwicklung der Diskussion um Eigenrechte der Natur dar und ging dabei vor allem auf außereuropäische Umsetzungsbeispiele ein. Anschließend sprach sie über den Mehrwert von Eigenrechten sowohl in materieller, als auch in prozessualer Hinsicht und verband dies mit einer Einordnung in den bestehenden deutschen und europäischen Rechtsrahmen. Abschließend ging sie auf die praktische Umsetzung des Konzepts der Eigenrechte ein. Dies stellte auch den Schwerpunkt der später folgenden Diskussion dar. Im Anschluss präsentierte *Nina Lanzer* (UFZ Leipzig) einen Zeitschriftenbeitrag zu einem „heiklen Thema": „Erst kommt das Fressen, dann kommt die Moral? – Juristische und tierethische Betrachtungen zum Töten von Tieren nach dem TierSchG". Nach einer anschaulichen Einführung zur Tierethik präsentierte sie ihre Überlegungen zu der Auslegung eines zentralen Begriffs des TierSchG, namentlich dem „vernünftigen Grund". Die dabei erfolgte Verknüpfung von Tierethik und juristischer Abwägung bildete den Hintergrund für die abschließende Analyse der „Küken-Urteile" des BVerwG.

Im letzten Block des ersten Tages referierte zunächst *Maximilian Weinrich* (Universität Würzburg) über „Das Jagdrecht im System des Umweltrechts". Im seinem Fokus standen die Verknüpfungen des Jagdrechts mit anderen Bereichen des Umweltrechts wie dem Naturschutzrecht, etwa die „Scharniernormen" §§ 37, 39 BNatSchG. Ein Beispiel für das Nebeneinander von Jagd- und Naturschutzrecht sei dagegen der Umgang mit invasiven Arten. Darüber hinaus wurden auch Bezüge zu dem vorherigen Vortrag über das TierSchG deutlich. Abschließend wurde es interdisziplinär: Die Chemieingenieurin *Katrin Schindler* (Hochschule Darmstadt) sprach über ihr Dissertationsvorhaben „SDG 12: Herausforderung für den Chemiehandel". Ihre Darstellung der sozialwissenschaftlichen Methodik war für die anwesenden Juristen eine Bereicherung. Neben der gewählten Methodik thematisierte sie vor allem, ob die Herangehensweisen von Unternehmen der Chemiewirtschaft den chemikalienrechtlichen Anforderungen aus SDG12 hinreichend entsprachen. Mit der REACH- und der CLP-Verordnung wurden auch Bezüge zum europarechtlichen Rahmen deutlich.

Am Freitag eröffnete *Jennifer Ernst* (Universität Frankfurt) mit ihrem Vortrag über „International Regulation of Solar Radiation Management Techniques". Zunächst stellte sie den aktuellen Entwicklungsstand verschiedener Techniken vor, welche insgesamt dem Bereich des sogenannten Climate Engineering zugeordnet werden. Diese sollen perspektivisch einen Beitrag zur Abmilderung des Treibhauseffekts leisten können. Sodann widmete sie sich einzelnen rechtlichen Problemen, vor allem im Rahmen einer möglichen völkerrechtlichen Regulierung. Dabei zeigte sie Parallelen zur bereits umgesetzten Regulierung von Gentechnik und Atomkraft auf. Im Anschluss präsentierte *Aline Araripe* ihr Vorhaben mit dem Arbeitstitel „Habermas Discourse Theory in Environmental Law". Den Untersuchungsgegenstand bildeten aktuelle Fälle von Umweltschäden in ihrer Heimat Brasilien, von wo aus sie auch ihren Vortrag um fünf Uhr morgens Ortszeit hielt. Beispielhaft genannt wurden die Zerstörung des Amazonas-Regenwaldes sowie eine Ölkatastrophe. Sie stellte vor, wie sie mit Hilfe der „Theorie des kommunikativen Handelns" von Jürgen Habermas das Umweltrecht als dynamisches Gebilde untersuchen möchte, um Spannungsfelder zwischen den verschiedenen Akteuren aufzudecken, zu verstehen und aufzulösen. Neben dem nationalen Umweltrecht solle auch das Völkerrecht untersucht werden.

*Henrik Fischer* (UFZ Leipzig) stellt mit „Verursachergerechte Finanzierung der Reduktion von Nährstoffeinträgen in die Gewässer" ein wasserrechtliches Thema vor. Im Zentrum stand die Lösung des Konflikts zwischen Kosteneffizienz, Verursacherprinzip und Gleichheitsgrundsatz im Rahmen der Finanzierung der ökologischen Gewässerbewirtschaftung. Anhand eines fiktiven Beispielfalls mit mehreren Akteuren wie Städten und Landwirten zeigte er auf, dass die bisherige Finanzierungsregelung in Deutschland ein von der Wasserrahmenrichtlinie abweichendes Verständnis der Finanzierung hat. Mithin werde den rechtlichen Anforderungen nicht entsprochen. Sodann präsentierte er alternative Regelungsansätze und ging auf deren praktische Umsetzung ein. Den letzten Vortrag hielt *Janna Ringena* (Universität Münster) über ihr Dissertationsthema „Einwegkunststoff: Regulierung durch die EU – Umsetzungen in Deutschland und Frankreich". Sie stellte vor, wie sie die Eingliederung der Vorgaben der neuen, hochaktuellen Einwegkunststoffrichtlinie in das bestehende System des Abfall- und Kreislaufwirtschaftsrechts untersuchen möchte und ging dabei auf verschiedene Problemschwerpunkte ein, zum Beispiel die Verbrauchsminderung von Einwegkunststoffprodukten. Darüber hinaus ging sie in ihren Ausführungen auf die Methode des funktionalen Rechtsvergleichs ein, mit deren Hilfe sie Erkenntnisse über die Umsetzung der Richtlinie in Frankreich für ihr Vorhaben gewinnen möchte.

## 2. Methodikfragen und Austausch

Für die Zeit zwischen den einzelnen Vortragsblöcken hatte das Organisationsteam die Idee, den generellen Austausch zwischen den Teilnehmern zu fördern, indem diese in „Breakout-Sessions“ zufällig in kleinere Gruppen unterteilt wurden. Innerhalb dieser Kleingruppen fand bei Kaffee und Snacks sodann ein ungezwungener Austausch statt. Die Idee wurde gut aufgenommen und es entwickelten sich schnell Gespräche, ähnlich wie an einem Stehtisch auf einer ProKUR in den Jahren zuvor. In einer dieser „Breakout-Sessions“ erfolgte die Zuordnung nicht zufällig, sondern anhand der jeweiligen Dissertationsthemen. Damit bestand auch die Möglichkeit für die Nichtvortragenden sich über ihr Themengebiet mit Teilnehmern aus „ihrem Feld“ auszutauschen und wertvolle Anregungen zu erhalten sowie zu geben.

Der Austausch über promotionsthemenübergreifende Inhalte erfolgte strukturiert im Rahmen von sogenannten Werkstattgesprächen. Dafür wurden die Teilnehmer abhängig von ihrem Fortschritt in eine von drei Gruppen eingeteilt: Frühphase (Themenfindung und Recherche), Hauptphase (Text schreiben) und Abschlussphase (Finalisieren und Publizieren). Unter Anleitung und Moderation durch sehr weit fortgeschrittene und ehemalige Doktoranden konnten die Teilnehmer über Probleme und Lösungen ihrer spezifischen Arbeitsphase diskutieren. Die Ergebnisse wurden im Anschluss von den Gruppen im Plenum vorgestellt, sodass alle Teilnehmer davon profitieren konnten. Für die Anfangsphasen-Gruppe standen Finanzierung der Promotion, Themenfindung, Recherchetipps und der Umgang mit nicht-juristischen Forschungsmethoden im Vordergrund. Die Hauptphasen-Gruppen, aufgrund der hohen Teilnehmerzahl gab es zwei, beschäftigten sich vor allem mit dem Schreibprozess als solchem, der Motivation, der Arbeitsorganisation und den Möglichkeiten das Dissertationsthema auf Veranstaltungen vorzustellen. Die Abschlussphasen-Gruppe brachte in das Plenum ihre Überlegungen zum Übergang in das Referendariat beziehungsweise das Berufsleben, die Verteidigung sowie Veröffentlichungsstrategien ein. Die erstmalig angebotenen Werkstattgespräche stießen insgesamt auf breite Zustimmung.

Im Rahmen der Möglichkeiten fand auch ein digitales Rahmenprogramm statt. Die Teilnehmer erhielten bei einer virtuellen Stadtführung die Gelegenheit Münster aus einer neuen Perspektive kennenzulernen. Abends wurde ein kurzweiliges Pubquiz mit zufällig gemischten Teams angeboten, sodass die Möglichkeit bestand, sich auch in lockererer Atmosphäre kennenzulernen.

## 3. Ausblick

In einer „Zukunftsdiskussion“ am Donnerstag tauschten die Teilnehmer sich darüber aus, wie das Format „ProKUR“ zukünftig weitergeführt werden soll. Um die Sichtbarkeit, gerade für angehende Doktoranden, womöglich ohne direkte Anbindung an einen Lehrstuhl, zu erhöhen, sollen eine Internetseite sowie ein Account bei Twitter eingerichtet werden. So sollen auch Alumni dem Netzwerk erhalten bleiben können. Zudem sei an dieser Stelle auf einen Newsletter aus dem Umfeld der ProKUR hingewiesen, welcher aktuelle Veranstaltungen, Call for Papers und Stellenausschreibungen enthält. Entsprechende Informationen können dort aufgenommen werden, indem sie an *promovieren-im-umweltrecht@posteo.de* geschickt werden. Eine Anmeldung für den Newsletter ist per E-Mail an die zuvor genannte Adresse möglich.

Die diesjährige ProKUR war ein voller Erfolg. Die Herausforderungen, die das digitale Format mit sich brachte, wurden von den Organisatoren vorausschauend angegangen und zur größten Zufriedenheit der Teilnehmer gelöst. Neue technische Möglichkeiten wurden zielführend eingesetzt. Auch das Veranstaltungskonzept als solches wurde mit den „Werkstattgesprächen“ bereichernd weiterentwickelt. Die Konferenzreihe wird im nächsten Jahr mit der vierten ProKUR fortgesetzt. Ein Team von der Universität Bonn übernimmt die Organisation.

**Open Access.** Dieser Artikel wird unter der Creative Commons Namensnennung 4.0 International Lizenz veröffentlicht, welche die Nutzung, Vervielfältigung, Bearbeitung, Verbreitung und Wiedergabe in jeglichem Medium und Format erlaubt, sofern Sie den/die ursprünglichen Autor(en) und die Quelle ordnungsgemäß nennen, einen Link zur Creative Commons Lizenz beifügen und angeben, ob Änderungen vorgenommen wurden.
Die in diesem Artikel enthaltenen Bilder und sonstiges Drittmaterial unterliegen ebenfalls der genannten Creative Commons Lizenz, sofern sich aus der Abbildungslegende nichts anderes ergibt. Sofern das betreffende Material nicht unter der genannten Creative Commons Lizenz steht und die betreffende Handlung nicht nach gesetzlichen Vorschriften erlaubt ist, ist für die oben aufgeführten Weiterverwendungen des Materials die Einwilligung des jeweiligen Rechteinhabers einzuholen.
Weitere Details zur Lizenz entnehmen Sie bitte der Lizenzinformation auf http://creativecommons.org/licenses/by/4.0/deed.de.

Funding

Open access funding enabled and organized by Projekt DEAL.

